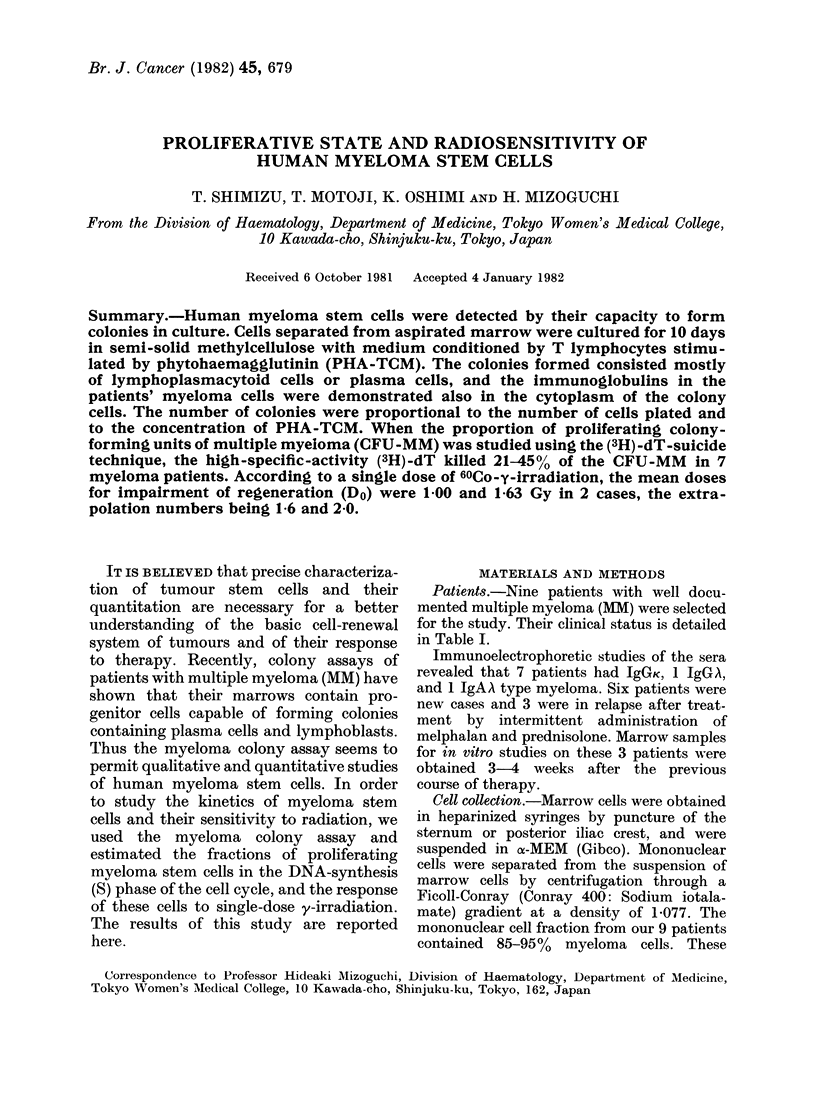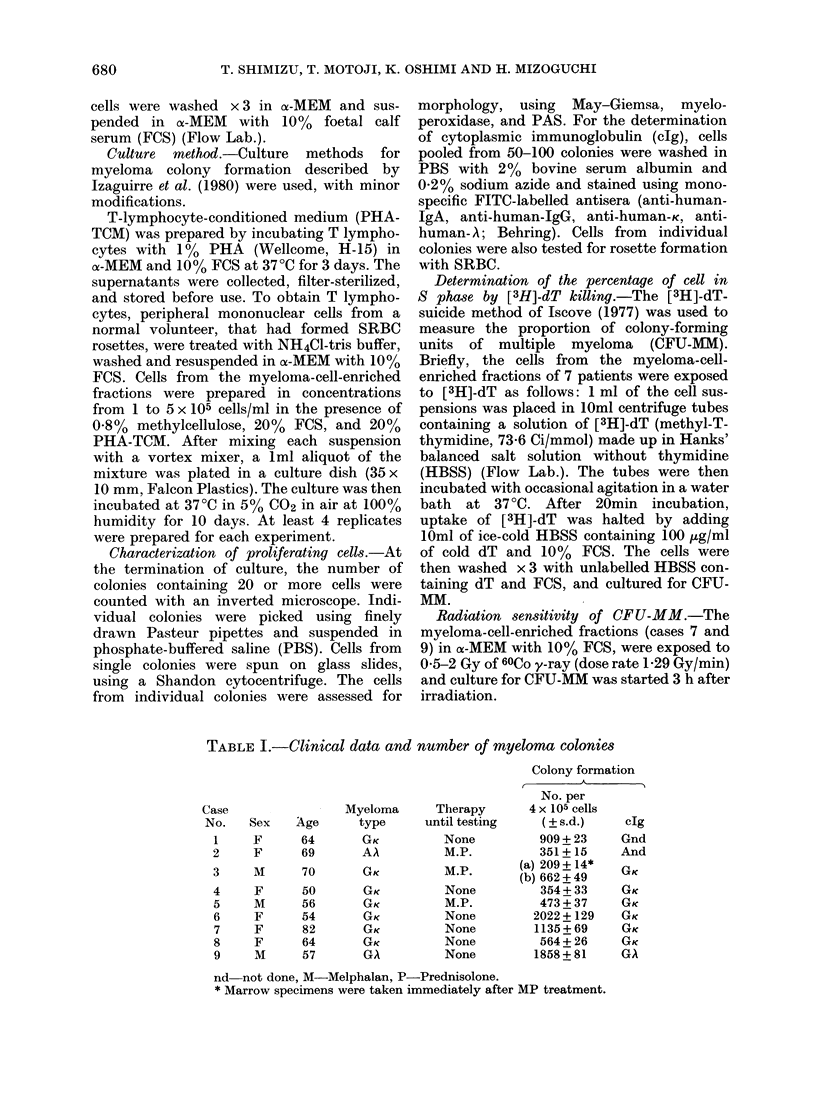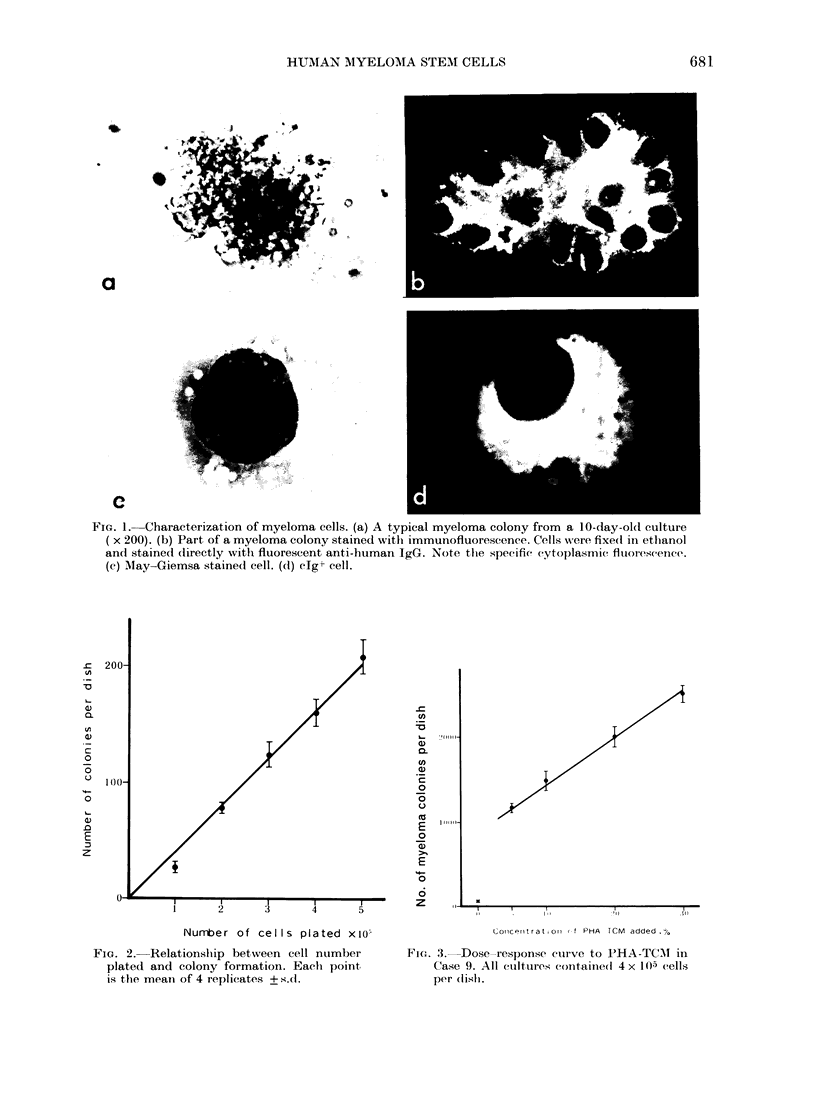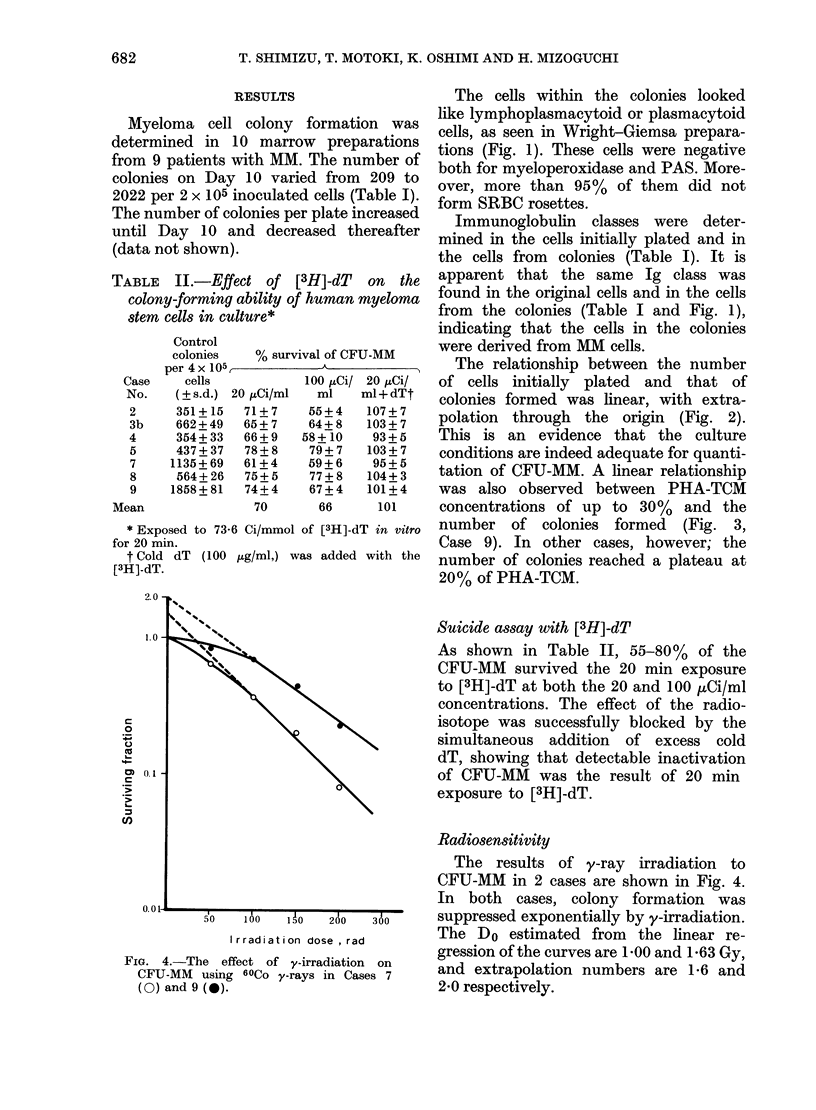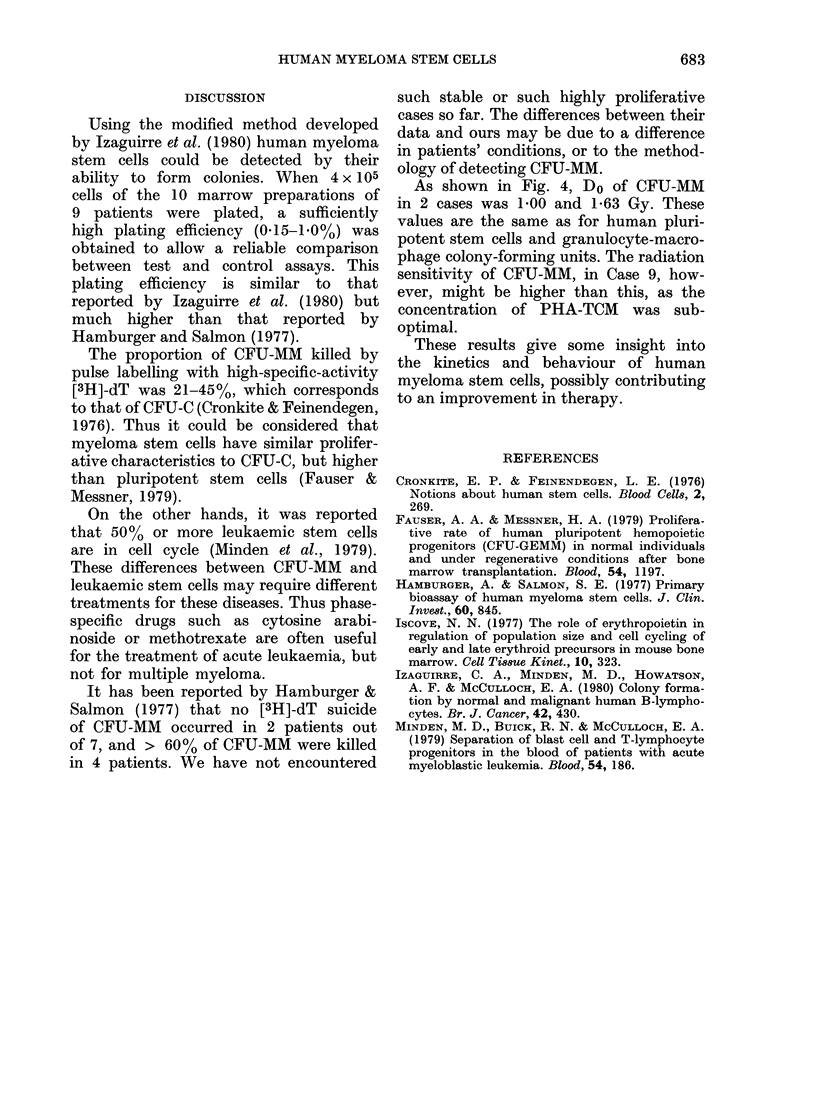# Proliferative state and radiosensitivity of human myeloma stem cells.

**DOI:** 10.1038/bjc.1982.108

**Published:** 1982-05

**Authors:** T. Shimizu, T. Motoji, K. Oshimi, H. Mizoguchi

## Abstract

**Images:**


*Br. J. Cancer* (1982) **45**, 679

### PROLIFERATIVE STATE AND RADIOSENSITIVITY OF HUMAN MYELOMA STEM CELLS

T. SHIMIZU, T. MOTOJI, K. OSHIMI AND H. MIZOGUCHI

*From the Division of Haematology, Department of Medicine, Tokyo Women's Medical College, 10 Kawada-cho, Shinjuku-ku, Tokyo, Japan*

Received 6 October 1981 Accepted 4 January 1982

**Summary.—Human myeloma stem cells were detected by their capacity to form colonies in culture. Cells separated from aspirated marrow were cultured for 10 days in semi-solid methylcellulose with medium conditioned by T lymphocytes stimulated by phytohaemagglutinin (PHA-TCM). The colonies formed consisted mostly of lymphoplasmacytoid cells or plasma cells, and the immunoglobulins in the patients' myeloma cells were demonstrated also in the cytoplasm of the colony cells. The number of colonies were proportional to the number of cells plated and to the concentration of PHA-TCM. When the proportion of proliferating colony-forming units of multiple myeloma (CFU-MM) was studied using the ($^3$H)-dT-suicide technique, the high-specific-activity ($^3$H)-dT killed 21–45% of the CFU-MM in 7 myeloma patients. According to a single dose of $^{60}$Co-$\gamma$-irradiation, the mean doses for impairment of regeneration ($D_0$) were 1·00 and 1·63 Gy in 2 cases, the extrapolation numbers being 1·6 and 2·0.**

IT IS BELIEVED that precise characterization of tumour stem cells and their quantitation are necessary for a better understanding of the basic cell-renewal system of tumours and of their response to therapy. Recently, colony assays of patients with multiple myeloma (MM) have shown that their marrows contain progenitor cells capable of forming colonies containing plasma cells and lymphoblasts. Thus the myeloma colony assay seems to permit qualitative and quantitative studies of human myeloma stem cells. In order to study the kinetics of myeloma stem cells and their sensitivity to radiation, we used the myeloma colony assay and estimated the fractions of proliferating myeloma stem cells in the DNA-synthesis (S) phase of the cell cycle, and the response of these cells to single-dose $\gamma$-irradiation. The results of this study are reported here.

### MATERIALS AND METHODS

*Patients.*—Nine patients with well documented multiple myeloma (MM) were selected for the study. Their clinical status is detailed in Table I.

Immunoelectrophoretic studies of the sera revealed that 7 patients had IgG$\kappa$, 1 IgG$\lambda$, and 1 IgA$\lambda$ type myeloma. Six patients were new cases and 3 were in relapse after treatment by intermittent administration of melphalan and prednisolone. Marrow samples for *in vitro* studies on these 3 patients were obtained 3—4 weeks after the previous course of therapy.

*Cell collection.*—Marrow cells were obtained in heparinized syringes by puncture of the sternum or posterior iliac crest, and were suspended in $\alpha$-MEM (Gibco). Mononuclear cells were separated from the suspension of marrow cells by centrifugation through a Ficoll-Conray (Conray 400: Sodium iotalamate) gradient at a density of 1·077. The mononuclear cell fraction from our 9 patients contained 85–95% myeloma cells. These

Correspondence to Professor Hideaki Mizoguchi, Division of Haematology, Department of Medicine, Tokyo Women's Medical College, 10 Kawada-cho, Shinjuku-ku, Tokyo, 162, Japan cells were washed ×3 in α-MEM and suspended in α-MEM with 10% foetal calf serum (FCS) (Flow Lab.).

*Culture method.*—Culture methods for myeloma colony formation described by Izaguirre *et al.* (1980) were used, with minor modifications.

T-lymphocyte-conditioned medium (PHA-TCM) was prepared by incubating T lymphocytes with 1% PHA (Wellcome, H-15) in α-MEM and 10% FCS at 37 °C for 3 days. The supernatants were collected, filter-sterilized, and stored before use. To obtain T lymphocytes, peripheral mononuclear cells from a normal volunteer, that had formed SRBC rosettes, were treated with $NH_4Cl$-tris buffer, washed and resuspended in α-MEM with 10% FCS. Cells from the myeloma-cell-enriched fractions were prepared in concentrations from 1 to $5 \times 10^5$ cells/ml in the presence of 0·8% methylcellulose, 20% FCS, and 20% PHA-TCM. After mixing each suspension with a vortex mixer, a 1ml aliquot of the mixture was plated in a culture dish (35 × 10 mm, Falcon Plastics). The culture was then incubated at 37 °C in 5% $CO_2$ in air at 100% humidity for 10 days. At least 4 replicates were prepared for each experiment.

*Characterization of proliferating cells.*—At the termination of culture, the number of colonies containing 20 or more cells were counted with an inverted microscope. Individual colonies were picked using finely drawn Pasteur pipettes and suspended in phosphate-buffered saline (PBS). Cells from single colonies were spun on glass slides, using a Shandon cytocentrifuge. The cells from individual colonies were assessed for morphology, using May–Giemsa, myeloperoxidase, and PAS. For the determination of cytoplasmic immunoglobulin (cIg), cells pooled from 50–100 colonies were washed in PBS with 2% bovine serum albumin and 0·2% sodium azide and stained using monospecific FITC-labelled antisera (anti-human-IgA, anti-human-IgG, anti-human-κ, anti-human-λ; Behring). Cells from individual colonies were also tested for rosette formation with SRBC.

*Determination of the percentage of cell in S phase by [$^3$H]-dT killing.*—The [$^3$H]-dT-suicide method of Iscove (1977) was used to measure the proportion of colony-forming units of multiple myeloma (CFU-MM). Briefly, the cells from the myeloma-cell-enriched fractions of 7 patients were exposed to [$^3$H]-dT as follows: 1 ml of the cell suspensions was placed in 10ml centrifuge tubes containing a solution of [$^3$H]-dT (methyl-T-thymidine, 73·6 Ci/mmol) made up in Hanks' balanced salt solution without thymidine (HBSS) (Flow Lab.). The tubes were then incubated with occasional agitation in a water bath at 37 °C. After 20min incubation, uptake of [$^3$H]-dT was halted by adding 10ml of ice-cold HBSS containing 100 μg/ml of cold dT and 10% FCS. The cells were then washed ×3 with unlabelled HBSS containing dT and FCS, and cultured for CFU-MM.

*Radiation sensitivity of CFU-MM.*—The myeloma-cell-enriched fractions (cases 7 and 9) in α-MEM with 10% FCS, were exposed to 0·5–2 Gy of $^{60}$Co γ-ray (dose rate 1·29 Gy/min) and culture for CFU-MM was started 3 h after irradiation.

TABLE I.—*Clinical data and number of myeloma colonies*

| Case No. | Sex | Age | Myeloma type | Therapy until testing | Colony formation: No. per $4 \times 10^5$ cells (±s.d.) | Colony formation: cIg |
|---|---|---|---|---|---|---|
| 1 | F | 64 | Gκ | None | 909 ± 23 | Gnd |
| 2 | F | 69 | Aλ | M.P. | 351 ± 15 | And |
| 3 | M | 70 | Gκ | M.P. | (a) 209 ± 14*<br>(b) 662 ± 49 | Gκ |
| 4 | F | 50 | Gκ | None | 354 ± 33 | Gκ |
| 5 | M | 56 | Gκ | M.P. | 473 ± 37 | Gκ |
| 6 | F | 54 | Gκ | None | 2022 ± 129 | Gκ |
| 7 | F | 82 | Gκ | None | 1135 ± 69 | Gκ |
| 8 | F | 64 | Gκ | None | 564 ± 26 | Gκ |
| 9 | M | 57 | Gλ | None | 1858 ± 81 | Gλ |

nd—not done, M—Melphalan, P—Prednisolone.

* Marrow specimens were taken immediately after MP treatment.

FIG. 1.—Characterization of myeloma cells. (a) A typical myeloma colony from a 10-day-old culture ( x 200). (b) Part of a myeloma colony stained with immunofluorescence. Cells were fixed in ethanol and stained directly with fluorescent anti-human IgG. Note the specific cytoplasmic fluorescence. (c) May–Giemsa stained cell. (d) $cIg^+$ cell.

FIG. 2.—Relationship between cell number plated and colony formation. Each point is the mean of 4 replicates ±s.d.

FIG. 3.—Dose–response curve to PHA-TCM in Case 9. All cultures contained $4 \times 10^5$ cells per dish.

### RESULTS

Myeloma cell colony formation was determined in 10 marrow preparations from 9 patients with MM. The number of colonies on Day 10 varied from 209 to 2022 per $2 \times 10^5$ inoculated cells (Table I). The number of colonies per plate increased until Day 10 and decreased thereafter (data not shown).

TABLE II.—*Effect of [³H]-dT on the colony-forming ability of human myeloma stem cells in culture**

| Case No. | Control colonies per $4 \times 10^5$ cells ($\pm$s.d.) | % survival of CFU-MM 20 μCi/ml | 100 μCi/ml | 20 μCi/ml + dT† |
|---|---|---|---|---|
| 2 | 351 ± 15 | 71 ± 7 | 55 ± 4 | 107 ± 7 |
| 3b | 662 ± 49 | 65 ± 7 | 64 ± 8 | 103 ± 7 |
| 4 | 354 ± 33 | 66 ± 9 | 58 ± 10 | 93 ± 5 |
| 5 | 437 ± 37 | 78 ± 8 | 79 ± 7 | 103 ± 7 |
| 7 | 1135 ± 69 | 61 ± 4 | 59 ± 6 | 95 ± 5 |
| 8 | 564 ± 26 | 75 ± 5 | 77 ± 8 | 104 ± 3 |
| 9 | 1858 ± 81 | 74 ± 4 | 67 ± 4 | 101 ± 4 |
| Mean | | 70 | 66 | 101 |

* Exposed to 73·6 Ci/mmol of [³H]-dT *in vitro* for 20 min.

† Cold dT (100 μg/ml,) was added with the [³H]-dT.

FIG. 4.—The effect of γ-irradiation on CFU-MM using $^{60}Co$ γ-rays in Cases 7 (○) and 9 (●).

The cells within the colonies looked like lymphoplasmacytoid or plasmacytoid cells, as seen in Wright–Giemsa preparations (Fig. 1). These cells were negative both for myeloperoxidase and PAS. Moreover, more than 95% of them did not form SRBC rosettes.

Immunoglobulin classes were determined in the cells initially plated and in the cells from colonies (Table I). It is apparent that the same Ig class was found in the original cells and in the cells from the colonies (Table I and Fig. 1), indicating that the cells in the colonies were derived from MM cells.

The relationship between the number of cells initially plated and that of colonies formed was linear, with extrapolation through the origin (Fig. 2). This is an evidence that the culture conditions are indeed adequate for quantitation of CFU-MM. A linear relationship was also observed between PHA-TCM concentrations of up to 30% and the number of colonies formed (Fig. 3, Case 9). In other cases, however; the number of colonies reached a plateau at 20% of PHA-TCM.

### *Suicide assay with [³H]-dT*

As shown in Table II, 55–80% of the CFU-MM survived the 20 min exposure to [³H]-dT at both the 20 and 100 μCi/ml concentrations. The effect of the radioisotope was successfully blocked by the simultaneous addition of excess cold dT, showing that detectable inactivation of CFU-MM was the result of 20 min exposure to [³H]-dT.

### *Radiosensitivity*

The results of γ-ray irradiation to CFU-MM in 2 cases are shown in Fig. 4. In both cases, colony formation was suppressed exponentially by γ-irradiation. The $D_0$ estimated from the linear regression of the curves are 1·00 and 1·63 Gy, and extrapolation numbers are 1·6 and 2·0 respectively.

### DISCUSSION

Using the modified method developed by Izaguirre *et al.* (1980) human myeloma stem cells could be detected by their ability to form colonies. When $4 \times 10^5$ cells of the 10 marrow preparations of 9 patients were plated, a sufficiently high plating efficiency (0·15–1·0%) was obtained to allow a reliable comparison between test and control assays. This plating efficiency is similar to that reported by Izaguirre *et al.* (1980) but much higher than that reported by Hamburger and Salmon (1977).

The proportion of CFU-MM killed by pulse labelling with high-specific-activity [$^3$H]-dT was 21–45%, which corresponds to that of CFU-C (Cronkite & Feinendegen, 1976). Thus it could be considered that myeloma stem cells have similar proliferative characteristics to CFU-C, but higher than pluripotent stem cells (Fauser & Messner, 1979).

On the other hands, it was reported that 50% or more leukaemic stem cells are in cell cycle (Minden *et al.*, 1979). These differences between CFU-MM and leukaemic stem cells may require different treatments for these diseases. Thus phase-specific drugs such as cytosine arabinoside or methotrexate are often useful for the treatment of acute leukaemia, but not for multiple myeloma.

It has been reported by Hamburger & Salmon (1977) that no [$^3$H]-dT suicide of CFU-MM occurred in 2 patients out of 7, and $>$ 60% of CFU-MM were killed in 4 patients. We have not encountered such stable or such highly proliferative cases so far. The differences between their data and ours may be due to a difference in patients' conditions, or to the methodology of detecting CFU-MM.

As shown in Fig. 4, $D_0$ of CFU-MM in 2 cases was 1·00 and 1·63 Gy. These values are the same as for human pluripotent stem cells and granulocyte-macrophage colony-forming units. The radiation sensitivity of CFU-MM, in Case 9, however, might be higher than this, as the concentration of PHA-TCM was sub-optimal.

These results give some insight into the kinetics and behaviour of human myeloma stem cells, possibly contributing to an improvement in therapy.

### REFERENCES

CRONKITE, E. P. & FEINENDEGEN, L. E. (1976) Notions about human stem cells. *Blood Cells*, **2**, 269.

FAUSER, A. A. & MESSNER, H. A. (1979) Proliferative rate of human pluripotent hemopoietic progenitors (CFU-GEMM) in normal individuals and under regenerative conditions after bone marrow transplantation. *Blood*, **54**, 1197.

HAMBURGER, A. & SALMON, S. E. (1977) Primary bioassay of human myeloma stem cells. *J. Clin. Invest.*, **60**, 845.

ISCOVE, N. N. (1977) The role of erythropoietin in regulation of population size and cell cycling of early and late erythroid precursors in mouse bone marrow. *Cell Tissue Kinet.*, **10**, 323.

IZAGUIRRE, C. A., MINDEN, M. D., HOWATSON, A. F. & MCCULLOCH, E. A. (1980) Colony formation by normal and malignant human B-lymphocytes. *Br. J. Cancer*, **42**, 430.

MINDEN, M. D., BUICK, R. N. & MCCULLOCH, E. A. (1979) Separation of blast cell and T-lymphocyte progenitors in the blood of patients with acute myeloblastic leukemia. *Blood*, **54**, 186.